# Serum Antibodies against *Helicobacter pylori* Neutrophil Activating Protein in Carriers of IL-4 C-590T Genetic Polymorphism Amplify the Risk of Gastritis and Gastric Cancer

**DOI:** 10.18869/acadpub.ibj.21.5.321

**Published:** 2017-09

**Authors:** Yeganeh Talebkhan, Mohsen Doozbakhshan, Samaneh Saberi, Maryam Esmaeili, Najmeh Karami, Nazanin Mohajerani, Afshin Abdirad, Mahmoud Eshagh Hosseini, Azin Nahvijou, Mohammad Ali Mohagheghi, Marjan Mohammadi

**Affiliations:** 1 HPGC Group, Department of Medical Biotechnology, Biotechnology Research Center, Pasteur Institute of Iran, Tehran, Iran; 2 Cancer Institute, Tehran University of Medical Sciences, Tehran, Iran; 3 Department of Gastroenterology, Amiralam Hospital, Tehran University of Medical Sciences, Tehran, Iran; 4 Cancer Research Center, Tehran University of Medical Sciences, Tehran, Iran

**Keywords:** Biomarkers, Genetic Polymorphism, Recombinant

## Abstract

**Background:**

Gastric cancer arises, mainly, on an inflammatory background. *Helicobacter pylori* neutrophil activating (HP-NAP) protein functions as a potent pro-inflammatory mediator. Similarly, IL-4 plays a critical role in the inflammation pathway, the levels of which are altered by C to T transition at position -590 in its promoter region. Here, we have aimed to assess the risk of gastritis and gastric cancer in the co-presence of these two inflammation modulating mediators.

**Methods:**

Gastritis (n=58) and gastric cancer (n=31) patients were evaluated and compared with *H. pylori*-positive asymptomatic controls (n=46), for serum antibodies against recombinant HP-NAP and IL-4 C-590T single nucleotide polymorphism using immunoblotting and PCR-RFLP, respectively. Multivariable logistic regression, adjusting for age, gender and ethnicity, was used for data analysis.

**Results:**

In terms of susceptibility to gastritis, seropositivity to HP-NAP projected a risk impact of 4.62 fold (OR=4.62, 95% CI=1.50-14.22), which when present in IL-4 -590 T carriers augmented the risk up to 9.7 fold (OR=9.70, 95% CI=2.06-45.69). A similar pattern, but of a stronger magnitude, occurred for the risk of gastric cancer, which was estimated at 9.07 fold (OR=9.07, 95% CI=1.99-42.0) for HP-NAP-seropositive subjects and was drastically amplified (OR=33.64, 95% CI=2.06-548.68), when double-positive (HP-NAP seropositive/IL-4 -590 T carrier) subjects were examined against double negatives (HP-NAP seronegative/IL-4 -590 CC).

**Conclusion:**

Our preliminary data indicate that serum antibodies against HP-NAP represent a state of risk, which is further exacerbated in IL-4 -590 T carriers. These biomarkers, if validated in larger prospective studies, can be used to screen for gastric cancer susceptibility

## INTRODUCTION

*Helicobacter pylori* infect nearly half of the world population, the majority of which remain asymptomatic. However in a small fraction (2-3%), infection leads to gastric cancer[1]. According to a recent study in a low-risk population, the transition of infected individuals through the so-called Correa cascade[2] increases their risk of developing gastric cancer, from 0.3% in those with normal mucosa; to 1.1% in gastritis patients; to 2% in atrophic gastritis; to 2.5% in intestinal metaplasia; and finally up to 5.2% in those with dysplasia[1]. There are a multitude of factors originating from the pathogen, host, and environment as well as their interactions, which come together to create grounds for the development of gastric cancer.

*H. pylori* produce a diverse repertoire of virulence factors. Amongst its highly immunogenic and pro-inflammatory antigens is the HP-NAP protein, known for its activation of neutrophils[3]. HP0243 is the gene encoding the 17-kDa subunit, which oligomerizes into the 150-kDa dodecameric structure of HP-NAP with a hollow internal core[4]. This conserved gene is differentially expressed amongst different *H. pylori* strains[4-6]. The most studied function for HP-NAP is recruitment and activation of neutorphils and monocytes and subsequent production of reactive oxygen intermediates[4,7], mediated by the activation of phagocytic NADPH-oxidase. A repertoire of other diverse functions has also been attributed to this protein which includes: (1) DNA binding[8] and protection from oxidative damage[6,9], (2) adhesion to mucins and mucosal surfaces[10], (3) iron-binding capability (up to 500 atoms)[11], (4) urease-independent acid resistance[12], and (5) immune activation with a pro-Th1 and anti-Th2 modulation and adjuvanticity[3,13-18] and has been demonstrated in different disease models. Host serum antibodies against HP-NAP are variably present in different populations[19-22] and have been associated with the risk of gastrointestinal complications including gastric cancer[23,24]. IL-4, on the other hand, takes precedence in its immune-modulating function and Th2 polarizing capacity. The Th1/Th2 balance and its pro- and anti-inflammatory downstream effects, although seemingly contradictory, are both documented in the gastric carcinogenic process[25,26]. The most frequent genetic alteration in the IL-4 gene occurs at position -590 in its promoter region. The C/T polymorphism at -590 position (rs 2243250) is associated with altered levels of IL-4 expression[27]. In parallel, the prevalence of gastric cancer is controversially reported to be associated with this genetic polymorphism[28-30].

In order to address the synergistic risk impact of these two inflammation-modulating mediators, we have assessed the independent and joint presence of serum antibodies to HP-NAP (originating from the pathogen) and IL-4 -590C/T SNP (originating from the host) in gastritis and gastric cancer patients, in comparison with *H. pylori*-positive asymptomatic controls.

## MATERIALS AND METHODS

### Production of recombinant HP-NAP

Amplification of HP-NAP gene fragment from *H. pylori* genomic DNA was carried out by PCR using the following forward (5′-GTCATATGAAAACATTT GAAATTTTAAAAC-3′) and reverse (5′-GTCTCGA GAGCCAAATGG-3′) primers, under the following conditions: one cycle of initial denaturation (95°C, 5 min), followed by 30 cycles of 95°C (1 min), 50°C (30 s), 72°C (1 min) and terminated by one cycle of final extension (72°C, 5 min). The amplified PCR product was cloned into pTZ57R T-vector (Promega, USA) and transformed into *E. coli* TOP10F’ strain (Invitrogen, USA). *NdeI/XhoI*-digested pET23a expression vector (Invitrogen, USA) was used for subcloning and transformation into *E. coli* BL21 (DE3) strain (Invitrogen, USA). Restriction digestion and partial sequencing were used to confirm the identity of the cloned gene fragment. Protein expression was induced by 0.5 mM IPTG during 4 hours of culture in LB broth. Western blotting using anti-6X His tag antibody (Roche, USA) and pooled *H. pylori*-positive and -negative sera were used to confirm the identity of the recombinant protein.

### Subjects

Our study population included 135 subjects categorized as follows: A) gastric cancer patients (n=31: age range=59.4±11.6, M:F=21:10; Cancer Research Center, Tehran, Iran), B) gastritis patients (n=58: age range=56.5±9.4, M:F=30:28; Amiralam Hospital, Tehran, Iran), and C) *H. pylori*-positive asymptomatic volunteers (n=46: age range=52.9±12.2, M:F=13:32; routine diagnostic laboratories, Tehran, Iran). Subjects under the age of 35 years (from all groups), those with peptic ulcers (from Group B), and those with dyspepsia (from Group C) were initially excluded. *H. pylori* infection was determined by serology. The demographic information of our study population is presented in Table 1. Blood samples were collected for serology and isolation of mononuclear cells. Gastric specimens were collected from patients undergoing gastric surgery or endoscopy for determination of gastric histopathology. The patient demographic information, including age, gender, and ethnicity was collected via personal interview (Table 1). The ethnicities of subjects were categorized as Fars (Persian) or non-Fars (Turk, Lor, Kurd, Gilaki, Mazani, etc.). Data and sample collections were carried out according to the protocols approved by the National Committee on Ethical Issues in Medical Research, Ministry of Health and Medical Education of Iran; Ref No. 315. A written informed consent was provided by every participant.

**Table 1:** Demographic characteristics of the study population

Characteristics	Gastric Cancer (%) (n=31)	Gastritis (%) (n=58)	Controls (%) (n=46)	*P* values	

				Gastric Cancer *vs.* Controls	Gastritis *vs.* Controls
**Age range**					
Mean ± SD	59.4 ± 11.6	56.5 ± 9.4	52.9 ± 12.2	0.027	0.144
**Gender**					
Male	67.9	51.4	26.7	0.001	0.025
Female	32.1	48.6	73.3		
**Ethnicity**					
Fars	19.2	27	57.8	0.001	0.007
Non-Fars	80.8	73	42.2		
**Inflammation** (*Grade)*					
0	-	2.7	-	-	-
I	-	43.2	-		
II	-	21.6	-		
III	-	18.9	-		
IV	-	13.5	-		
**Atrophy *(Stage)***					
0	-	59.5	-	-	-
I	-	29.7	-		
II	-	8.1	-		
III	-	2.7	-		
IV	-	0	-		
**Tumor *(Subsite)***					
Cardia	42.9	-	-	-	-
Non-Cardia	53.6	-	-		
Mixed	3.5	-	-			
**Tumor *(Subtype)***					
Intestinal	50	-	-	-	-
Diffuse	29.2	-	-		
Signet ring cell	4.2	-	-		
Mixed	8.3	-	-		
Other	8.3	-	-		
**Tumor *(Stage)***					
IA	4.6	-	-	-	-
IB	14.7	-	-		
II	19.0	-	-		
IIIA	38.1	-	-		
IIIB	19.0	-	-		
IV	4.6	-	-		
**Tumor *(Grade)***					
Poor	34.8	-	-	-	-
Moderate	30.4	-	-		
Well	34.8	-	-		
**Serum anti-NapA antibodies**					
Negative	41.9	60.3	76.1	0.003	0.02
Positive	58.1	39.7	23.9		
**IL4 -590 Genotype**					
CC	45.2	37.9	71.7	0.02	0.0008
T carrier (CT+TT)	54.8	62.1	28.3		

### Gastric histopathology

Gastric specimens were obtained from the proximal (corpus), middle (incisura angularis), and distal (antrum) stomach according to the modified Sydney’s method[31]. Paraffin-embedded, H&E (hematoxylin and eosin)-stained sections were evaluated for the grade of gastric inflammation (grades 0-IV) and the stage of gastric atrophy (stages 0-IV), according to the OLGA method of classification[32] (Table 1). Gastric adenocarcinoma patients were categorized based on tumor location (cardia, noncardia, or mixed), histological subtype (intestinal/diffuse/mixed)[33], grade of differentiation (well/moderate/poor)[34], and stage of metastasis[35] (Table 1).

### Immunoblotting

Patients’ sera were evaluated for the presence of IgG antibodies against rHP-NAP by Western blotting. Briefly, rHP-NAP was run on 15% SDS-PAGE, transferred to nitrocellulose membrane and blocked overnight in 2% skim milk. Diluted patient sera were applied to the membranes. The membranes were reacted with HRP-conjugated anti-human IgG (DAKO, Denmark) and developed by 3,3′-diaminobenzidine (Sigma, USA).

### IL-4-590 C/T single nucleotide polymorphism

In order to detect the C to T transition at position -590 in the IL-4 promoter region, PCR-RFLP was carried out using the indicated forward (5’-TAAACTTGGGAGAACATGGT -3’) and reverse (5’-TGGGGAAAGATAGAGTAATA-3’) primers. The PCR conditions were as follows: one cycle of initial denaturation (94°C, 3 min), followed by 35 cycles of 94°C (30 s), 53.5°C (30 s), 72°C (30 s) and terminated by one cycle of final extension (72°C, 3 min), which amplified a 195-bp fragment of the gene. The PCR products were digested with 1 unit of *Ava*II restriction enzyme (Fermentas, Lithuania) at 37°C for 3 hours. The digested products were then run on 3% agarose gel, and the resulting bands were interpreted as follows: CC: 195 bp, TT: 175 bp+20 bp, and CT: 195 bp+175 bp+20 bps. Subjects carrying one (CT) or two (TT) mutant alleles were categorized as T carriers (CT+TT).

### Statistical analysis

Data analysis was conducted using STATA software (v.12). Statistical associations were calculated by Chi-square (χ^2^) and fisher’s exact tests. Multivariable regression analysis, adjusting for potential confounders (age, gender, and ethnicity), calculated the odd ratios (OR) and 95% confidence intervals (95% CI). Probability (*P)* values were considered statistically significant, if less than 0.05.

## RESULTS

### Production of the recombinant HP-NAP

**Fig. 1:**
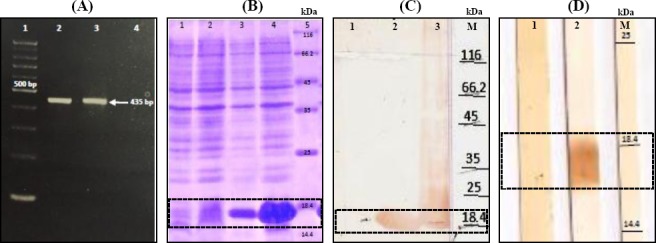
Cloning, expression, and identity confirmation of recombinant HP-NAP. (A) PCR amplification of napA gene. Lane 1, 100 bp DNA marker; Lanes 2 and 3, *H. pylori* napA gene; Lane 4, negative control. (B) SDS-PAGE analysis of the expressed HP-NAP protein. Lane 1, pET23a (before induction); Lane 2, pET23a (after induction); Lane 3, pET23a-*napA* (before induction); Lane 4, pET23a-*napA* (after induction); Lane 5, the protein size marker. (C) Western blotting using anti-6X His tag antibody. Lane 1, BI (before induction); Lane 2, Sup (bacterial culture supernatant after induction); Lane 3, Pellet (bacterial culture pellet after induction); Lane M, the protein size marker. (D) Western blotting using pooled patient sera. Lane 1, *H. pylori*-negative; Lane 2,: *H. pylori*-positive; Lane M, the protein size marker.

The PCR-amplified 435-bp gene fragment encoding rHP-NAP (Fig. 1A) was cloned and subcloned into the linearized pTZ57R and pET23a vectors, respectively. *NdeI/XhoI* restriction digestion of the recombinant vector released the cloned fragment and therefore confirmed its identity. The cloned fragment was sequenced, and the resulting information was deposited in the GenBank (accession No.: KC616343.1). BLAST analysis of the sequenced coding region confirmed its identity at the genetic level. Determination of its sequence homology with other HP-NAP gene sequences was carried out by BioEdit and Clustal X. The result exhibited 95-100% nucleotide similarity between our (Iranian) gene sequence and those of *H. pylori* strains from different geographic locations, as well as that of J99 reference strain (98.3%, data not shown). IPTG induction of BL21 (DE3) *E. coli* cells harboring HP-NAP-pET28a resulted in the expression of the ~17-kDa recombinant HP-NAP protein (Fig. 1B). The identity of the recombinant protein was confirmed at two levels by Western blotting using anti-6X His tag antibody (Fig. 1C) and pooled *H. pylori*-positive sera (Fig. 1D).

### Risk of gastritis in HP-NAP sero-positive, IL-4 -590T carriers

**Fig. 2:**
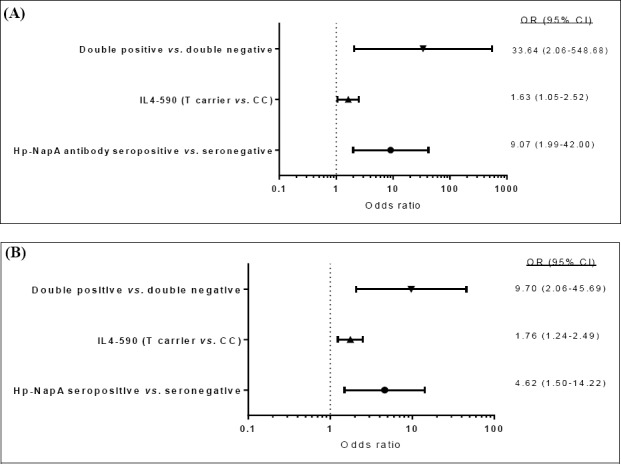
Forest plots presenting the odds ratio and 95% confidence intervals for the evaluated biomarker. Odds of developing (A) gastritis or (B) gastric cancer, in reference to *H. pylori*-positive asymptomatic controls. Double positive, HP-NAP seropositive/C-590T carriers; double negative HP-NAP seronegative/IL-4 -590 CC. All calculations were adjusted for age, gender, and ethnicity.

Gastritis patients were evaluated for the grade of inflammation and stage of atrophy (Table 1). Every subject was diagnosed with various grades of gastric inflammation, about 40% of whom were presented with gastric atrophy. We evaluated our study population for serum antibodies against rHP-NAP and found 39.7% of gastritis patients as seropositive, compared to only 23.9% of the *H. pylori*-positive asymptomatic controls (*P*=0.02). Multivariable regression analysis, adjusting for age, gender, and ethnicity, identified HP-NAP seropositive subjects at 4.62 fold (OR=4.62, 95% CI=1.50-14.22) greater risk of gastritis (Fig. 2A), in reference to HP-NAP seronegative subjects.

In order to detect IL-4 C-590T single nucleotide polymorphism (SNP), genomic DNA was extracted from each subject and underwent PCR-RFLP. The population was categorized into subjects with wild (CC) or mutant (T carrier=CT+TT) genotypes. A statistically significant majority of gastritis (62.1%, *P*=0.0008) patients were either heterozyote (CT) or homozygote (TT) for IL-4 C-590T SNP, as compared to only 28.3% of *H. pylori*-positive asymptomatic controls. Multivariable regression analysis identified IL-4-590 T carriers (CT and TT) at a moderately increased risk of gastritis ([OR=1.76, 95% CI=1.24-2.49], Fig. 2A). Joint examination of the above mentioned two variables placed double positive (HP-NAP-seropositive/-590T carrier) subjects at a 9.7 fold (OR=9.70, 95% CI=2.06-45.69) amplified risk of gastritis, in reference to double negatives (HP-NAP seronegative/IL-4 -590 CC; Fig. 2A).

### Risk of gastric cancer in HP-NAP sero-positive, IL-4 -590T carriers

Gastric cancer patients were mostly diagnosed with tumors of the noncardia anatomic subsite (53.6%) and intestinal histologic subtype (50%; Table 1). The above biomarker analysis was also carried out for gastric cancer patients. The significant majority of whom (58.1%) were seropositive for HP-NAP, as compared to *H. pylori*-positive asymptomatic controls (23.9%, *P*=0.003). The projected risk impact for gastric cancer was nearly double (OR=9.07, 95% CI=1.99-42.0) that of gastritis (OR=4.62, 95% CI=1.50-14.22) in HP-NAP-seropositive subjects, in reference to HP-NAP seronegative subjects (Fig. 2B).

Overall, 54.8% of gastric cancer patients carried the IL-4 -590T SNP, which was significantly higher than that of *H. pylori*-positive asymptomatic controls (28.3%, *P=*0.02), which again recognized IL-4 -590 T carriers (CT and TT) at a moderately increased risk of gastric cancer [(OR=1.63, 95% CI=1.05-2.52), Fig. 2B).

Co-presence of the above two markers, however, drastically multiplied the odds ratio and placed double-positive (HP-NAP-seropositive/-590T carrier) subjects at more than 30 fold (OR=33.64, 95% CI=2.06-548.68) increased risk of gastric cancer, as compared to double-negative (HP-NAP seronegative/IL-4 -590 CC) subjects (Fig. 2B).

## DISCUSSION

Gastric inflammation is a hallmark of *Helicobacter pylori* infection, from which other *H. pylori*-associated gastrointestinal complications, including gastric cancer are believed to arise[36]. Therefore, any variables that exacerbate the gastric inflammatory response could theoretically potentiate the risk of gastric cancer. In this study, we have examined the co-presence of two inflammation-modulating factors, one originating from the infecting organism (HP-NAP) and the other from the host (IL-4C-590T SNP).

HP-NAP is recognized for its ability to activate and recruit neutrophils[3]. It can also serve to protect *H. pylori* from the toxic damages inflicted by the inflammatory cells[8,9], as well as to cultivate the bacterium with the resulting by-products[11]. The immunogenic properties of HP-NAP have repeatedly nominated it as a putative vaccine candidate[7]. Its function in switching the immune response from Th2 to Th1 type has been employed as an adjuvant in Th2-type diseases such as asthma[37], and *in situ* regression of tumors such as that of the bladder[38]. Its dual pathogenic/therapeutic functions[3,39] have created the paradox as to whether its expression in the *H. pylori*-infected subjects works to exacerbate or protect against the induced gastrointestinal complications, and whether it can be used as a screening biomarker.

Here, we have examined host HP-NAP-specific antibodies to partly address the above paradox. Our findings, from a case-control study in Iran, demonstrated that subjects with serum antibodies against HP-NAP were at higher risk of developing gastric inflammation (4.6 fold) and to a greater extent, gastric cancer (9.1 fold). Long and colleagues[24] measured the levels of HP-NAP-specific antibodies in various clinical groups of *H. pylori*-infected patients in China and reported a statistically significant step-wise elevation of serum antibodies from healthy subjects (sero-positivity: 27.7%, OD: 0.65±0.18), to chronic gastritis (sero-positivity: 85.7%, OD: 0.89±0.14), to gastric cancer (sero-positivity: 97.7%, OD: 1.01±0.24) patients. Using multiplex serology, Song *et al*.[23] evaluated serum antibodies specific to *H. pylori* CagA and non-CagA factors for their association with gastric adenocarcinoma in Sweden. Among the non-CagA factors, they found HP-NAP-specific antibodies to project a risk impact of 2.6 fold (OR=2.6, 95% CI: 1.7-4.0) for GAC, in general and 3.4 fold (OR=3.4, 95% CI: 2.1-5.4) for noncardia GAC. Similarly, Liu *et al*.[40], using multivariate logistic regression, adjusting for potential confounders (age, gender, smoking, and alcohol drinking), found Chinese HP-NAP seropositive subjects at 9.2 (OR=9.2, 95% CI: 1.96-43.14) fold increased risk of gastric cancer, in reference to the controls. The most convincing evidence comes from a recent report[41] on a collection of eight cohorts from China, Japan, and Korea (1447 cases and 1801 controls), in which HP-NAP seropositivity placed subjects at a 1.45 (1.26-1.68) fold magnified risk for gastric cancer.

On the other hand, the role of functional single nucleotide gene polymorphisms in inflammation-modulating cytokines have long been associated with the risk of diseases arising from chronic inflammatory backgrounds, including epithelial malignancies such as gastric cancer. Here, we have targeted IL-4 C-590T SNP, since previous reports from Asian populations have demonstrated a risk impact for gastric cancer in IL-4 -590T carriers, in reference to the wild genotype. However, this notion was not confirmed by other investigators[29,30]. In our study, using multivariable logistic regression, having adjusted for the potential confounders of age, gender, and ethnicity, revealed an estimated 76 and 63% increased risk of gastritis and gastric cancer in IL-4 -590T carriers, respectively.

Having jointly assessed the above two variables, we found a synergistic amplification of risk, when both variables were present in one individual. Such that double-positive subjects were found at 9.7 fold and 33.6 fold amplified risk of gastritis and gastric cancer, respectively. To our knowledge, there are no previous reports of joint examination of these two biomarkers as risk biomarkers for gastritis or gastric cancer. The main limitation of this study is the small sample sizes, which create a low power (as evident by the broad confidence intervals), when assessing the risk of gastritis or gastric cancer in double positives versus double negatives. Nevertheless, if our results are validated in larger populations, they nominate joint assessment of these two biomarkers as a simple non-invasive, readily accessible tool for screening gastric cancer-at-risk populations. Therefore, larger case-control studies rising from prospective cohorts are recommended to re-examine this hypothesis.
